# Cellular Stress in the Pathogenesis of Muscular Disorders—From Cause to Consequence

**DOI:** 10.3390/ijms21165830

**Published:** 2020-08-13

**Authors:** Alexander Mensch, Stephan Zierz

**Affiliations:** Department of Neurology, Martin-Luther-University of Halle-Wittenberg, 06120 Halle (Saale), Germany; stephan.zierz@uk-halle.de

**Keywords:** myopathy, muscular dystrophy, pathomechanism, oxidative stress, ER-stress, unfolded protein response, integrated stress response, mitochondrial stress response, hypoxia

## Abstract

Cellular stress has been considered a relevant pathogenetic factor in a variety of human diseases. Due to its primary functions by means of contractility, metabolism, and protein synthesis, the muscle cell is faced with continuous changes of cellular homeostasis that require rapid and coordinated adaptive mechanisms. Hence, a prone susceptibility to cellular stress in muscle is immanent. However, studies focusing on the cellular stress response in muscular disorders are limited. While in recent years there have been emerging indications regarding a relevant role of cellular stress in the pathophysiology of several muscular disorders, the underlying mechanisms are to a great extent incompletely understood. This review aimed to summarize the available evidence regarding a deregulation of the cellular stress response in individual muscle diseases. Potential mechanisms, as well as involved pathways are critically discussed, and respective disease models are addressed. Furthermore, relevant therapeutic approaches that aim to abrogate defects of cellular stress response in muscular disorders are outlined.

## 1. Introduction

The reaction of cells to internal or external changes of homeostasis by means of a concerted cellular stress response is a key aspect of cell survival and maintenance of cellular integrity [1]. Corresponding to this assumption, deregulation of the cellular stress response has been identified as a relevant feature in the pathogenesis of numerous human diseases. Among others, the cellular stress response has been implicated to partake a pivot role in cardiovascular, metabolic, and neurodegenerative disorders [2,3,4].

In context of neuromuscular disorders, a variety of recent studies suggest a relevant involvement of the cellular stress response in the pathogenesis of several neuromuscular diseases. In this respect, the devastating motor neuron disorder amyotrophic lateral sclerosis (ALS) is by far the best studied disease model regarding a deregulated cellular stress machinery as a relevant pathogenetic mechanism. This is partly due to the fact that the first gene identified to be involved in familial forms of ALS was the *SOD1* gene [5]. This gene encodes for superoxide dismutase 1, a catalase that is a key factor in the cellular reaction to oxidative stress [6]. Since then, mutations in several other factors involved in stress response were linked to familial forms of ALS, as such *TARDBP* (TDP-43, transactive response DNA binding protein 43 kDa), *FUS* (fused in sarcoma), *VCP* (valosin containing protein), and *TIA1* (Tia1 cytotoxic granule-associated RNA binding protein) [7]. Consequently, the cellular stress response was identified as a major component in the pathogenesis not only in familial but also sporadic forms of ALS (e.g., reviewed in Reference [8]). Apart from ALS, the cellular stress response has been suggested to be involved in several forms of both hereditary and acquired peripheral neuropathy, with familial amyloid polyneuropathy (FAP) and diabetic polyneuropathy as two prominent examples [9,10].

However, although the impact of the cellular stress response on homeostasis of the central and peripheral nervous system has been extensively studied, comparably little attention has been paid to the muscle as primary effector. This is intriguingly surprising, as the muscle appears to be an organ that is particularly exposed to changes of cellular homeostasis. Based on this assumption, a prone susceptibility to defects of the cellular stress response can be assumed. Accordingly, several recent publications have addressed the topic of cellular stress response in the context of muscular disorders.

Thus, the aim of this review was to describe the recent advances regarding the role of cellular stress in the pathogenesis of muscle diseases. Based on findings applicable to all muscular disorders, disease-specific findings are described, and emerging clinical consequences are discussed. 

## 2. The Cellular Stress Response in Context of Muscle Cells

The muscle is the largest organ in the human body with 30–40% of the human body mass being held by skeletal muscles [11,12]. To fulfill its primary function by means of motion, postural stabilization, and breathing, the muscle tissue requires extensive and continuous supplementation of energy, as well as a thorough adaption to changes in metabolism and oxygen consumption [13,14]. In this regard, muscle oxygen uptake can account for up to 90% of the whole-body oxygen uptake during exercise [15]. In addition, an elevated oxidative capacity, as well as higher mitochondrial DNA content in cardiac and skeletal muscle have been proposed as compared to other human tissues [16]. Hence, an extensive production of reactive oxygen species (ROS) in course of muscle cell metabolism can be assumed. Furthermore, the muscle tissue serves as one of the major sites to regulate human body metabolism apart from the liver [14]. Again, this entails rapid and profound changes in cellular metabolism that require an advanced regulation machinery to prevent cellular damage. The skeletal muscle contains 50–75% of all the body’s proteins and thus demands a tight and multimodal regulation of protein synthesis, as well as an adaptive response to misfolded proteins both in health, as well as muscle injury [17]. 

Taking these considerations into account, a prone vulnerability of the muscle to a deregulation of the cellular stress response is immanent. Accordingly, several recent studies have identified respective deteriorations in the pathophysiology of muscular disorders. While the understanding of the role of the cellular stress response in muscle diseases is still beginning to emerge, some basic principles have become apparent that are noteworthy to address.

Depending on the type of cellular stress, the cell initiates a distinct response that orchestrates a complex machinery of specific factors with the aim to overcome the respective stressor. Some of these mechanisms are comparably specific to the type of stress applied and thus termed accordingly (e.g., unfolded protein response, oxidative stress response). However, all initiated pathways converge into a set of shared response mechanisms called Integrated Stress Response (ISR) that provides general mechanisms to restore cellular homeostasis [18].

**Figure 1:**
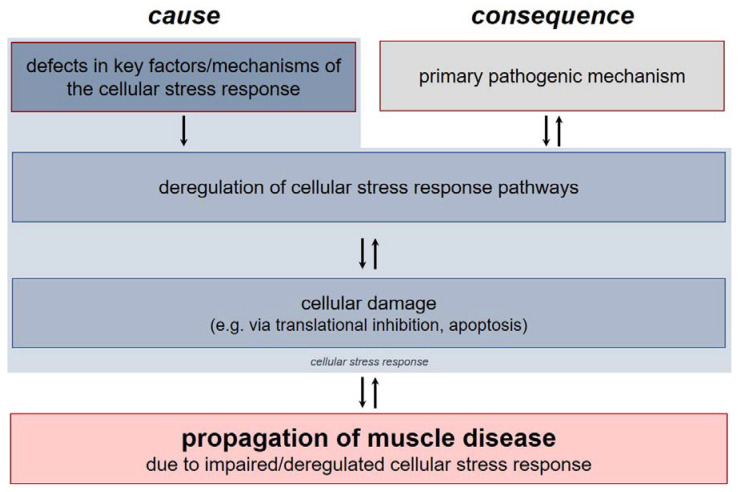
General principles of stress response-related pathogenesis in muscular disorders. While there are some muscular disorders that emerge from defects in key factors or mechanisms of the cellular stress response (‘cause’), in most muscle diseases, the deregulation of cellular stress response pathways arises as result of another pathogenic mechanism (‘consequence’).

As for muscular disorders, there are two general principles to be considered (Figure 1). First, the respective muscle disease may lead to an overactivation or deregulation of the cellular stress response and subsequently, by means of a vicious circle, to a propagation of cellular damage due to the transcriptional and post-transcriptional changes initiated. In this scenario, deregulation of the cellular stress response must be considered the consequence of the specific muscle disease. On the other hand, several recent studies have reported a disturbance in key mechanisms or factors of the cellular stress response as causative pathogenetic mechanism. In this pathogenetic model, defects of the cellular stress response are the primary cause of the muscle disease.

To further elucidate these considerations, the specific changes of particular stress response pathways in the context of the individual muscular disorders are addressed in detail in the following sections. Although there is a relevant interaction between different forms of cellular stress, and most muscular disorders tend to deregulate the cellular stress response via several mechanisms, the individual types of cellular stress responses are discussed separately for intelligibility reasons. A comprehensive summary of muscular disorders with deregulated cellular stress response is given in Table 1.

## 3. Endoplasmic Reticulum (ER)-Stress and Unfolded Protein Response

The involvement of ER-stress and the unfolded protein response in the pathogenesis of several muscle diseases is perhaps the best studied mechanism regarding cellular stress in muscular disorders. The skeletal muscle contains a large and complex endoplasmic reticulum (ER) network termed sarcoplasmic reticulum (SR). As in all other cell types, its key function is the concerted synthesis of new proteins in terms of translation, folding, processing, and trafficking [101]. Apart from protein synthesis, regulation of the cellular calcium homeostasis is another major function of the ER. As changes in cellular calcium levels are of particular importance for muscle contraction, the SR appears to be more specialized in this manner compared to other cell types [102]. 

Disruption of ER-function leads to the initiation of the unfolded protein response (UPR) in order to restore proper protein synthesis and reduce the amount of misfolded proteins [103]. One major activator of the UPR is the aggregation of misfolded proteins in the ER-lumen, that is primarily sensed via three transmembrane proteins: RNA-dependent protein kinase-like ER eukaryotic translation initiation factor 2 alpha kinase (*PERK*), inositol-requiring protein 1 (*IRE1*), and activating transcription factor-6 (*ATF6*). Under physiological conditions, these transmembrane sensors are bound to the chaperone heat shock 70 kDa protein 5 (*HSPA5*, also known as BiP/glucose-regulating protein 78) and thus remain inactivated. Accumulation of misfolded proteins leads to the dissociation of *HSPA5* and binding to the misfolded proteins, resulting in an activation of *PERK*, *IRE1*, and *ATF6* [17].

The activation of PERK mediates several responses, including global translational inhibition via phosphorylation of eukaryotic translation initiation factor 2α (*eIF2α*) and translation of activating transcription factor-4 (*ATF4*). *ATF4* leads to transcriptional activation of a set of UPR-related factors, including chaperones, the pro-apoptotic transcription factor C/EBP homologous protein (*CHOP*), as well as the regulatory phosphatase growth arrest and DNA damage-inducible protein (*GADD34*) [103,104,105,106]. *IRE1*, on the other hand, promotes splicing of the X-box-binding protein 1 (*XBP1*)-mRNA, leading to increased amounts of ER-chaperones and other factors related to proper protein folding [107,108]. *ATF6* is translocated to the nucleus after cleavage in the Golgi apparatus, where it, among other functions, acts synergistically with XBP1 yield protein folding capacity [109,110]. A major role of ER-stress and UPR has originally been ascribed to cell types with high secretory activity (e.g., pancreatic β-cells) that show a continuous and extensive protein synthesis. However, some evidence suggests that even under physiological conditions skeletal muscle may also use the ER protein folding capacity to near its limits [111].

Besides the genuine excess of unfolded proteins, several other mechanisms that activate the UPR have been identified. As described above, the ER is the main storage of intracellular calcium and thus tightly involved in cellular calcium metabolism. Thus, disturbance of the cellular calcium homeostasis is a potent driver of ER-stress, especially as several heat shock proteins and chaperones, as well as the protein folding itself, are calcium-depended [112]. Furthermore, changes in membrane composition by means of an altered content of specific lipids have been identified to trigger ER-stress, as well as UPR [113,114]. Notably, there is a vivid reciprocal interaction between ER-stress and oxidative stress. Altered oxidative capacity has been identified to induce ER-stress, which in turn induces ROS production in both ER and mitochondria [115].

**Figure 2:**
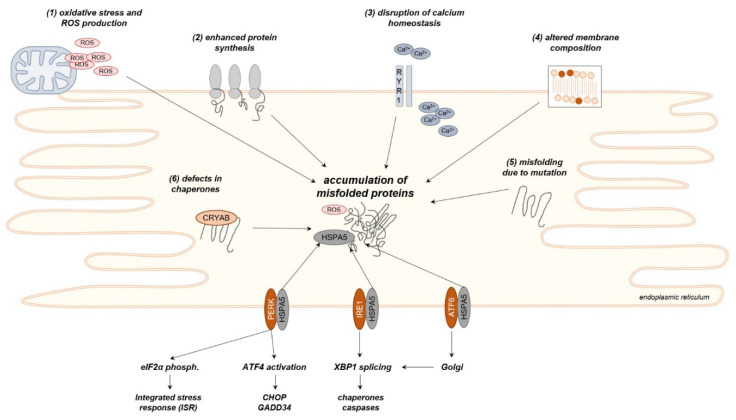
Schematic representation of relevant sources and mechanisms of endoplasmic reticulum (ER)-stress in muscular disorders. A variety of conditions result in the accumulation of misfolded proteins. HSPA5 dissociates from PERK, inositol-requiring protein 1 (IRE1), and activating transcription factor-6 (ATF6) in order to bind to the misfolded proteins, leading to the activation of several downstream pathways. *ROS – Reactive Oxygen Species*.

Irrespective of the underlying source, perturbation or continuous overactivation of the ER-stress response results in cellular damage and finally apoptosis [116]. Consistently, deregulation of ER-stress and the UPR has been identified as one major component of several muscular diseases. Figure 2 summarizes major sources and mechanisms of ER-stress in muscle diseases, while findings in individual muscular disorders are presented below.

### 3.1. ER-Stress in Inflammatory Myopathies

Idiopathic inflammatory myopathies (IIM) are a heterogenous group of acquired myopathies that are characterized by chronic muscle inflammation, as well as progredient muscular weakness. Based on clinical, histological, and serological findings, IIM is classified into several subtypes: polymyositis, dermatomyositis/overlap syndrome with myositis, necrotizing myopathy, and inclusion body myositis [117]. Apart from the damage mediated by local tissue infiltration of respective immune cells, increasing evidence is gained that non-immune-dependent mechanisms contribute to the pathogenesis of IIM. Among others, this assumption was mainly based on a study providing evidence that upregulation of MHC-I molecules on the surface of muscle cells (a prominent feature of all IIMs) in an immunodeficient mouse strain was able to induce a severe myopathy with histologic features resembling IIM histopathology [118]. Of note, several recent studies have reported upregulation of key factors of the ER stress-/UPR-pathway in IIM, suggesting a deregulation of the ER stress response as pathogenetic mechanism in IIM. 

Although a deregulated cellular ER stress response has been proposed in basically all subtypes of IIM [37,38,39], inclusion body myositis has been of keen interest in this regard. Sporadic inclusion body myositis (sIBM) is the most common muscular disorder in patients above 50 years of age and clinically characterized by progressive muscular weakness with prominent involvement of finger flexors and quadriceps femoris, as well as progressive dysarthria and dysphagia. Besides a variable degree of inflammatory cells, the histopathologic correlates of the disease include irregularly shaped vacuoles, as well as intracytoplasmic inclusion bodies. The evident inclusion bodies contain a variety of proteins, including amyloid-β (A-β), phosphorylated tau (p-tau), and several other proteins associated with Alzheimer disease [119]. Based on the finding that these aggregates are among others positive for Congo Red staining, it has been proposed that they contain un-/misfolded proteins that may contribute to inclusion body myositis (IBM)-pathogenesis [40]. In accordance, several studies have identified markers of ER-stress and activation of UPR (e.g., calnexin, *HSPA5*, *GRP170*) to be upregulated and to colocalize with the cytoplasmic protein aggregates, both in patient samples and experimental mouse models of IBM [41,42,43,44,45]. However, the mechanisms leading to misfolding of the afore mentioned proteins is still controversially discussed. Besides the influence of inflammatory pathways on protein folding and degradation pathways [46,47], several studies have suggested an innate deregulation of chaperone and proteasomal activity in sIBM [37,39,45]. Of note, there are diverging results regarding the role of MHC-I expression as driver of protein misfolding and deregulation of UPR pathways [39,118]. Furthermore, a recent study has proposed changes in calcium homeostasis as relevant source of protein misfolding and aggregation in sIBM [48]. Additionally, experimental evidence has been gained suggesting enhanced autophagy pathways and mTOR-signalling to contribute to sIBM vacuolated pathology [49]. As the deregulation of ER-stress-/UPR-pathways and aggregation of unfolded proteins are a potent activator of autophagy, deregulation of ER-stress may further contribute to sIBM pathogenesis via autophagy induction.

Irrespective of the mechanisms leading to the deregulation of ER-stress and UPR in sIBM, there have been therapeutic approaches aiming to restore proper protein folding and reverse protein aggregation. Of particular interest in this regard is arimoclomol, a drug that induces the heat-shock response and thus holds the potential to restore protein homeostasis. While arimoclomol was able to improve IBM pathology and muscle strength both in in vitro studies, as well as in an in vivo mouse model for inclusion body myopathy (mutant valosin-containing protein), the efficacy endpoint was not met in a subsequently conducted phase 1 clinical trial. The small sample size (8 patients in verum-treated arm) and short duration of the treatment (4 months) have been proposed as potential reasons for the fail to show efficacy [120]. As results were nonetheless promising, a larger phase 2/3 study including 150 patients has been initiated that is expected to complete in 2021 (ClinicalTrials.gov Identifier: NCT02753530).

### 3.2. ER-Stress in Muscular Dystrophies

Muscular dystrophies are a phenotypically and genotypically heterogeneous group of inherited muscular disorders that share common histopathologic features, including degeneration/necrosis, regeneration, and endo- and perimysial fibrosis, as well as increased adipose tissue [121]. Causative gene mutations leading to individual muscular dystrophies reflect a broad spectrum of cellular functions, including extracellular matrix proteins, members of the dystrophin-glycoprotein complex, nuclear envelope proteins, and mitochondrial membrane proteins, amongst others [122]. Beside the pathogenetic consequences arising from individual gene mutations, additional aggrieving mechanisms have been identified that are not causally related to the gene product primarily affected in the individual muscular dystrophy. In this regard, ER-stress has been identified as a relevant source of muscular damage in several muscular dystrophies. The individual muscular dystrophies that show a deregulated ER-stress response/UPR can be found in Table 1. As for the underlying cause of ER-stress in muscular dystrophies, there has been a variety of suggestions. Hypotheses discussed include accumulation of misfolded protein due to the respective mutation in structural proteins and altered protein folding capacity because of oxidative stress and ROS production, as well as changes in calcium homeostasis. However, specific experimental evidence is missing to a great extent [13,17]. 

Dystrophinopathies are the most prevalent forms of muscular dystrophies. While Duchenne muscular dystrophy (DMD) is a devastating muscle disease leading to rapid progression of muscular weakness, early loss of ambulation and premature death, Becker muscular dystrophy (BMD) usually shows a milder phenotype. The causative *DMD* gene encodes for the dystrophin protein, a core component of the dystrophin-glycoprotein complex that connects the muscle cell to the extracellular matrix [123]. Due to its prone function in muscle cell structure, the primary pathogenetic mechanism appears to be a disintegration of muscle cell organization and reduced resistance to mechanical stress. However, several studies have provided evidence that ER-stress and the UPR might also be a relevant factor in dystrophinopathies. Upregulation of BiP, as well as several UPR-related factors (e.g., *PDI*, *PERK*, *XBP1*), has been identified both in patient samples, as well as mdx mice, a widely used mouse model to study DMD [20,21,22]. Regarding the source of ER-stress in DMD, oxidative stress and occurrence of ROS have been proposed as main inductors of ER-stress/UPR [20]. Furthermore, an impaired ER-mitochondria interaction and concomitant dysregulation of calcium homeostasis may further accentuate ER-stress response in DMD [22]. Of potential interest is a recent study showing that knockout of Caspase-12, a downstream effector of the UPR in mdx mice partially ameliorates muscle damage and restores muscle force, emphasizing the role of ER-stress in DMD pathology [21]. While there is growing evidence of a deregulation of ER-stress in DMD, sufficient information regarding BMD is missing. Thus, it remains elusive whether the findings of DMD also apply for BMD. However, it can be speculated that the maintenance of a (not fully functional) dystrophin protein in BMD may not activate ER-stress and UPR in a comparable manner as in DMD.

Apart from dystrophinopathies, upregulation of ER-stress/UPR related factors has been identified in several other forms of muscular dystrophy. A relevant dysregulation has been proposed in limb-girdle muscular dystrophies (LGMD) related to mutations in *FKRP* (LGMD2I/LGMD R9), as well as in *CAV3* (LGMD1C/RMD2). While in terms of *FKRP*-related LGMD2I upregulation of BiP, *CHOP*, and other UPR-related factors has been demonstrated both in patient samples, as well as in zebrafish, findings in CAV3-related LGMD1C solely rely on studies in mouse models [19,28,29]. Interestingly, dysregulation of ER-stress has been identified in Nonaka myopathy, a distal muscular dystrophy caused by mutation in the *GNE* gene. Nonaka myopathy is also called hereditary inclusion body myositis (hIBM) as it resembles clinical and histopathological features of sporadic inclusion body myositis. In correspondence to the findings in sIBM, upregulation of both BiP, as well as downstream regulators, including several caspases is observed in patients with Nonaka myopathy [23,24]. Thus, the co-occurrence of ER-stress/UPR in both Nonaka myopathy, as well as sIBM, strongly emphasizes the role of ER-stress for development of disease-specific pathology. Finally, activation of UPR has also been observed in Tibial muscular dystrophy, a late-onset distal muscular dystrophy related to mutations in the *TTN* gene encoding for the titin protein [30].

Of prone interest is the emerging evidence that mutations in several genes encoding for heat shock proteins are associated with the development of (neuro-)muscular disorders [25]. In particular, mutations in *BAG3* (BAG family molecular chaperone regulator 3), *CRYAB* (α-crystallin B chain), and *DNAJB6* (DnaJ homolog subfamily B member 6) are known to cause individual forms of muscular dystrophy [26,27,31,32]. Interestingly, desmin-positive cytoplasmic aggregates containing disrupted and misfolded myofibrillar proteins are a common histologic feature of these myopathies (myofibrillar myopathy) [124]. While *BAG3* primarily functions as co-factor in chaperone-assisted selective autophagy, *CRYAB* is a small heat shock protein predominantly expressed in heart and skeletal muscle, where it has been shown to ensure muscular integrity by binding to intermediate filaments in the Z-band region [125,126]. The small heat shock protein *DNAJB6* was recently identified as a relevant inhibitor of ER-stress [127]. The fact that mutations in genes whoseproducts promote proper protein folding and thus ensure protein homeostasis are causing a predominant muscular pathology may serve as further evidence for the importance of ER-stress in the pathogenesis of muscular disorders. Furthermore, they display an example of mutations in key factors of the cellular stress response as primary pathogenic mechanism of muscle diseases.

### 3.3. ER-Stress in Disturbance of Calcium Homeostasis

As mentioned before, dysregulation of intracellular calcium levels has been identified as relevant source of ER-stress with the highly specialized muscular SR being particularly susceptible. An important regulator of intrasarcoplasmic calcium levels is the ryanodine receptor 1, a calcium channel in the sarcoplasmic reticulum that is encoded by the *RYR1* gene. Mutations in *RYR1* cause a spectrum of muscular disorders that vary in onset, clinical phenotype, and histopathology, including central core myopathy and centronuclear myopathy, as well as malignant hyperthermia susceptibility [128]. Depending on the individual mutation, there is a heterogenous impact on intracellular calcium homeostasis. While some mutations seem to increase temperature-dependent SR calcium leak, mitochondrial dysfunction, and oxidative stress, others decrease SR calcium leak and do not seem to rely on oxidative stress as major driver of muscle degeneration. A recent study focusing on one of the most common mutation in *RYR1* (I4898T) that leads to decreased calcium permeation has identified ER-stress and UPR to be persistently upregulated, resulting in induction of pro-apoptotic pathways. Consistently, treatment with the chemical chaperone sodium 4-phenylbutyrate (4PBA) markedly improved muscle function in a mutation-specific mouse model [34]. 

Analogical results have been gained in a disease model of selenoprotein N-related myopathy (*SELENON*-myopathy), a clinical heterogenous muscle disease that shares common features with *RYR1*-myopathy. In *SELENON*-myopathy, a reduced sarcoplasmic calcium uptake is considered to induce ER-stress and UPR, which in turn further impinges calcium uptake to the ER via an ER oxidoreductin 1 (*ERO1*)-dependent mechanism [33]. 

### 3.4. ER-Stress in Metabolic Myopathies

Metabolic myopathies are a heterogenous group of hereditary muscle diseases that emerge due to mutations in key enzymes of muscle metabolism. Potentially impaired metabolic pathways include glycolysis, glycogenolysis, fatty acid transport, and oxidation, as well as energy production in mitochondria [129]. One important member of myopathies is Glycogen storage disease type II (Pompe disease), an autosomal recessive muscle disorder characterized by abnormal glycogen storage due to deficiency of the lysosomal acid alpha-glucosidase (*GAA*). Typical symptoms include progressive proximal muscle weakness, cardiac affection, as well as restrictive ventilatory defects [130]. While progressive storage of intralysosomal glycogen is the primary pathogenetic mechanism in Pompe disease, emerging evidence suggests that excessive accumulation of autophagic vacuoles is a key factor in disease propagation, especially in patients that are resistant to enzyme replacement therapy [131,132]. In this concern, ER-stress was suggested to be a major inductor of autophagy via activation of p38 mitogen-activated protein kinase in patient derived fibroblasts. Treatment with the pharmacological chaperone *N*-butyl-deoxynojirimycin (NB-DNJ) was able to significantly reduce autophagic vacuoles, again illustrating the tight connection of ER-stress and autophagy pathways [35].

A recent publication focusing on Lipin1 deficiency, a metabolic myopathy characterized by recurrent episodes of rhabdomyolysis and increased lipid storage in muscle cells, has identified ER-stress as primary driver of myodegeneration [36]. Lipin 1 is a phosphatidate phosphatase that provides substrates needed for the synthesis of phospholipids and triacylglycerol [133]. At first glance, it appears enigmatic that defects in lipid synthesis are primarily leading to predominant activation of the UPR pathway. However, changes in ER-membrane composition sensed by *IRE1* is known to be a potent source of ER-stress [134,135]. Correspondingly, *IRE1* is found to be relevantly upregulated in a mouse model of Lipin 1 deficiency. Treatment with the chemical chaperone tauroursodeoxycholic acid (TUDCA) was able to ameliorate both muscle weakness, as well as lipid droplet accumulation, in Lipin 1 deficient mice, again emphasizing the importance of ER-stress in the pathogenesis of Lipin 1 deficiency [36].

### 3.5. ER-Stress in Myasthenia Gravis

Myasthenia gravis is an acquired autoimmune disease characterized by progressive activity-aggravated muscular weakness. The primary cause are autoantibodies to elements of the neuromuscular junction (e.g., anti-acetylcholine receptor autoantibody, antimuscle-specific kinase autoantibody, among others) resulting in perturbed excitation and thus reduced muscular contraction [136]. Although complement-mediated destruction of the postsynaptic neuromuscular junction membrane is considered to be the key pathogenic mechanism in acetylcholine receptor-autoantibody positive myasthenia gravis, ER-stress seems to relevantly add to propagation of the muscle weakness. While one study reported an upregulation of BiP in muscle biopsies of patients with myasthenia, recently published evidence suggests that ER-stress promotes acetylcholine receptor endocytosis and thus contributes to disease progression [50,51]. 

## 4. Oxidative Stress Response

In general, oxidative stress is considered as imbalance of prooxidant and antioxidant mechanisms leading to a dysregulation of the cellular homeostasis towards a prooxidative environment [137]. Under normal conditions, there is a tightly balanced equilibrium of prooxidative and antioxidative factors, that ensures proper functioning of metabolic pathways, as well as cellular integrity. However, if the amount of prooxidative factors exceed the antioxidative capacity, there is a shift towards a prooxidative environment and thus occurrence of oxidative stress [138]. In this regard, reactive oxidative species (ROS), such as superoxide anion (O2^−^) and hydrogen peroxide (H_2_O_2_), are considered to be the major drivers of a prooxidative state. As consequence of the accumulation of these oxidative factors, there is a direct and irreversible damage of a variety of macromolecules, including proteins and membrane lipids, as well as DNA, resulting in a progressive promotion of oxidative tissue damage [139]. While oxidative stress was initially assumed to be primarily harmful and destructive, recent works suggest that there is an innate need for basal levels of oxidative stress as it ensures proper function of antioxidant pathways to guarantee cellular integrity [140]. 

Muscle tissue has been widely accepted as one of the organs that are particularly subjected to oxidative stress due to excessive generation of prooxidative factors. As mentioned above, there is a continuous demand of high energy levels and oxygen uptake to facilitate contraction. As for the cellular source of ROS production in muscle, there is a vivid debate. Irrespective of their individual contingent, electron leakage from the mitochondrial respiratory chain, as well as cellular NAD(P)H-oxidases, have been identified as the major sites of ROS generation (profoundly reviewed in Reference [141]). As mentioned before, there is a tight entanglement of oxidative stress and ER-stress by means of a vicious circle. While a thoroughly balanced redox state is mandatory for proper protein folding in the ER, protein folding is associated with the production of high amounts of ROS. Thus, deregulation of either the antioxidant system or protein folding is likely to have a profound impact on the other [142].

As result of oxidative stress, there is an orchestrated activation of redox-sensitive enzymes and transcription factors, leading to the induction of an integrated cellular antioxidant defense, as well as pro-apoptotic pathways, respectively [138]. While a comparably broad variety of signaling pathways was assumed to be involved in oxidative stress response, some effectors are considered to be of particular importance [143]. One of these effectors is Heme-regulated eIF2α kinase (*HRI*), a redox-sensitive kinase that is autophosphorylated via a heme-dependent mechanism and forms active heterodimers with Heat shock protein 90 (Hsp90) [144]. Upon its activation, the HRI/Hsp90 complex mediates eIF2α-phosphorylation and thus induces the integrated stress response (ISR) [18]. Another potent mediator of the oxidative stress response is the transcription factor Nuclear factor-erythroid 2-related factor 2 (Nrf2). Under physiological conditions, Nrf2 is subject of rapid ubiquitination by Kelch-like ECH-associated protein 1 (Keap1). However, changes in redox status towards an oxidative environment leads to modification of Keap1, resulting in the inability to ubiquitinate Nrf2 [145]. This results in increased Nrf2 protein levels in the nucleus, where Nrf2 activates stress-responsive genes (e.g., *GST*, *SOD1*) by binding to specific elements in the promotor region termed antioxidant-responsive elements (ARE) [146]. An additional central pathway of the antioxidative defense is the NFκB-pathway. NFκB is normally stored in the cytoplasm and kept inactivated due to binding of inhibitory proteins called inhibitor of nuclear factor kappa B (IκB). In response to oxidative stress, IκB is modified or degraded, thus allowing NFκB to enter the nucleus and promote the expression of several antioxidant target genes. However, due to broad range of genes activated via NFκB, evidence has been gained that there is also an activation of prooxidant factors via NFκB-signaling [147]. Apart from Nrf2 and NFκB, other important redox-sensitive transcription factors activated by oxidative stress include peroxisome proliferator-activated receptor gamma (PPARγ), forkhead box protein O (FOXO), activator protein 1 (AP-1), heat shock factor 1 (HSF1), and hypoxia-inducible factor 1-alpha (HIF-1α) [148]. 

While initial works focused on ROS-generation and oxidative stress during exercise under physiological conditions, increasing evidence has been gained that oxidative stress may be a relevant pathogenic mechanism in several muscle disorders. 

### 4.1. Oxidative Stress in Muscular Dystrophies

Like in terms of ER-stress, muscular dystrophies are perhaps the best studies disease models regarding oxidative stress in muscular disorders. An overview of muscular dystrophies in which the pathogenesis includes oxidative stress response is shown in Table 1. Again, most of these studies are focusing on dystrophinopathies by means of Duchenne muscular dystrophy (DMD) and Becker muscular dystrophy (BMD). A detailed description of both diseases may be found in Section 3. 

Early evidence indicative of a relevant disruption of antioxidant defense mechanisms in DMD was that muscle biopsies of both patients and mdx mice contained elevated amounts of oxidized lipids and proteins [52,53,54,55,56,57,58,59]. Of potential interest in this regard was a study showing that in mdx mice, accumulation of oxidized products preceded dystrophic changes in muscle biopsies taken from presymptomatic mice [60]. Furthermore, it has been found that knockout of central genes responsible for antioxidative defense results in histopathologic changes similar to dystrophinopathies, again illustrating the central role of oxidative stress in muscular dystrophies [61]. 

As for the source of oxidative stress in DMD, several mechanisms have been discussed. First, a marked reduction of neuronal nitric oxide synthase (nNOS), that has been identified as a member of the dystrophin-glycoprotein complex, is observed in dystrophinopathies [149,150]. As nNOS produces the highly reactive free radical nitric oxide (NO), that rapidly reacts with other ROS, a profound in change in cellular redox state as consequence of nNOS deficiency has been discussed. Supporting this hypothesis, muscle-specific expression of nNOS in mdx mice was able to relevantly abrogate the histopathology of dystrophinopathy [151]. Furthermore, the pronounced inflammatory response regularly observed in dystrophinopathies has been discussed as a major source of ROS, mainly through macrophage-induced free radical mediated cellular lysis [152]. 

**Figure 3:**
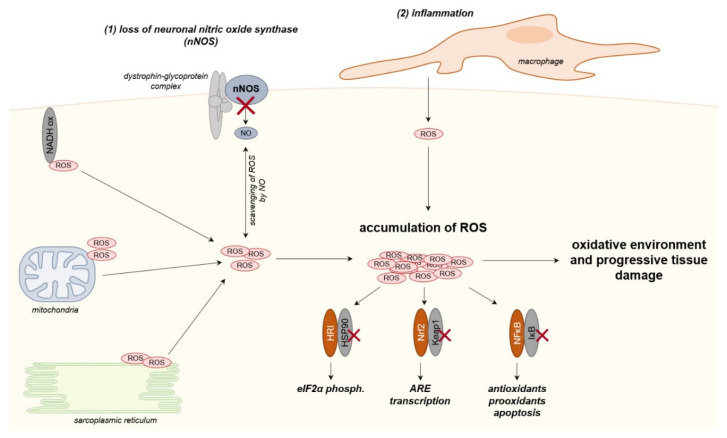
Oxidative stress in Duchenne muscular dystrophy (DMD). Due to the perturbation of the dystrophin-glycoprotein-complex, there is a loss of neuronal nitric oxide synthase (nNOS), which produces the scavenging radical nitric oxide (NO). Hence, there is a progressive accumulation of reactive oxygen species (ROS). Chronic inflammation may further yield ROS via macrophage-induced free radical mediated cellular lysis. As a result, several downstream pathways, including the Nuclear factor-erythroid 2-related factor 2 (Nrf2)-pathway and the NFκB-pathway, are initiated.

Whether oxidative stress alone is able to induce the pathogenic deterioration observed in dystrophinopathies is subject of ongoing debate, with most authors favoring a “two-hit model” meaning the necessity of a second pathogenic factor to develop DMD/BMD pathology [153,154]. However, irrespective of the relevance of oxidative stress for the initiation of DMD pathology, a relevant upregulation of the oxidative stress response has been observed in both patients, as well mdx mice, throughout disease progression. Evidence has been gained that both Nrf2-pathways and NFκB-pathways are highly upregulated in DMD. Interestingly, it appears that activation of both pathways is pronounced in early phases of the disease and declines with progression of dystrophy [155,156]. Figure 3 illustrates relevant mechanisms of oxidative stress-related muscular damage in DMD. 

As oxidative stress appears to be relevant pathogenic factor in DMD/BMD, there have been several attempts to induce a therapeutic strategy, mainly aiming to abrogate dysregulation of the antioxidant defense. Several antioxidants, including tocopherols (vitamin E) and selenium, as well as superoxide dismutase (SOD), have been used to treat patients with DMD but failed to show any improvements of muscular weakness [157,158,159]. Of note, most of the studies have included a small number of patients, the majority of them in advanced disease stages. Furthermore, it has been questioned whether the right antioxidants have been chosen and whether local antioxidant concentrations achieved in these trials are sufficient to relevantly influence the redox state in muscle tissue [154]. Nonetheless, the fact that treatment with antioxidants did not improve DMD pathology raises concerns whether oxidative stress is an essential part of DMD pathogenesis. In this regard, recently published studies using either deferoxamine or gold nanoparticles to treat mdx mice have yielded promising results [160,161]. Taken together, there is a need for both pre-clinical and clinical trials to evaluate the therapeutic potential of drugs aiming to reduce oxidative stress in DMD.

Apart from DMD, oxidative stress has been identified as a relevant factor contributing to pathogenesis in several other muscular dystrophies. One of them is Facioscapulohumeral muscular dystrophy (FSHD), a progressive muscular dystrophy primarily affecting facial, shoulder girdle, and upper arm muscles. FSHD is caused by complex genetic changes resulting from a failure to sufficiently suppress the *DUX4* gene, a pleiotropic transcription factor normally expressed during embryogenesis and repressed in adult specimen. The most common mutation leading to enhanced *DUX4*-expression is a deletion of regulatory DNA-elements next to the *DUX4*-locus, termed *D4Z4*-compression. This constellation is called FSHD1, while other mutations leading to *DUX4*-overexpression are classified as FSHD2 [162]. Several reports have demonstrated that upregulation of both oxidation products, as well as oxidative stress-related pathways (NFκB), are early events in FSDH pathogenesis that precede dystrophic changes in histology [63,64]. In this regard, evidence has been gained that there is a clear correlation between oxidative stress and the grade of muscular impairment [65]. Interestingly, a recent study reports an increased expression of *DUX4* upon application of oxidative stress in patient-derived induced pluripotent stem cells (iPSC), mainly driven by ATM serine/threonine kinase (*ATM*) [66]. Furthermore, there has been evidence that *DUX4*-expression itself induces oxidative stress leading to relevant defects in myotube formation, while antioxidant treatment was able to abrogate this effect [67]. Hence, a vicious circle of *DUX4*-expression and oxidative stress can be assumed that contributes to FSHD pathogenesis. Supporting this assumption, a double-blind randomized controlled clinical trial of antioxidant supplementation (vitamin C, vitamin E, zinc gluconate, and selenomethionine) has shown promising results regarding improvement of muscle strength upon antioxidant treatment in FSHD patients [163].

Another muscular dystrophy that shows profound activation of oxidative stress-induced pathways is *GNE*-associated myopathy, that has been discussed in terms of ER-stress above. Recently gained evidence suggests that oxidative stress contributes to the dystrophic changes and that treatment of *GNE*-deficient mice with the antioxidant N-acetylcysteine (NAC) was able to ameliorate *GNE* histopathology [68]. 

Finally, a relevant upregulation of oxidative stress and NFκB-signalling has been identified in dysferlinopathy (LGMD2B/LGMDR2), a muscular dystrophy arising from mutation in the *DYSF*-gene. Analyses of both patient-derived muscle biopsies, as well as *DYSF*-knockdown cells, identified a relevant increase of both oxidative stress and NFκB-pathway as a result of *DYSF*-deficiency, while the underlying mechanisms remain vastly elusive [62].

### 4.2. Oxidative Stress in Mitochondriopathies

As mentioned before, electron leakage of the mitochondrial respiratory chain (RC) is a major source of ROS production in muscle cells. Thus, it appears reasonable that defects of mitochondrial metabolism, especially in terms of an altered energy production via the RC may result in enhanced ROS production and thus induce profound changes of the cellular redox state. Mitochondriopathies are a heterogenous group of disorders that result from mutations in either nuclear or mitochondrial DNA (mtDNA) genes that encode for proteins exclusively located in the mitochondria. As virtually all organs rely on mitochondria to ensure proper energy supplementation, mitochondriopathies usually display dysfunction of multiple organ systems. However, several mitochondriopathies show a predominant muscular phenotype [164,165]. 

The individual muscle cells in most mtDNA-associated diseases, such as chronic progressive external ophthalmoplegia (CPEO), mitochondrial encephalomyopathy with lactic acidosis and stroke-like episodes (MELAS), and myoclonus epilepsy with ragged red fibers (MERRFs), show a varying grade of respiratory chain defects, depending on the individual mutational burden. The histological correlate commonly used to reflect this mosaicism is the coexistence of cells negative to cytochrome c oxidase (*COX*, complex IV of the respiratory chain) staining and *COX*-positive cells [165]. There has been emerging evidence that oxidative stress is a relevant contributor to pathogenesis in several mtDNA-associated diseases. In this regard, it has been demonstrated, that markers of oxidative stress and ROS production were elevated in cells with defects of mtDNA and that these changes appeared to rise with increasing mutational load of the cells [166,167,168]. Consistently, a recent study using single fiber proteomic analysis in muscle biopsies of patients with mtDNA-associated diseases found a robust upregulation of several antioxidant factors in *COX*-negative cells [69]. As a consequence of oxidative stress in mitochondria, dysregulation of several stress-induced signalling-pathways, including AMPK- and NFκB-signalling, have been identified [70,71]. While there is very little evidence regarding therapeutic options arising from oxidative stress in mtDNA-associated diseases, a pilot study using a combination of different antioxidants has gained promising results [72,73]. In contrast, the results of a recently published phase I/II clinical trial focusing on omaveloxolone as potent inductor of Nrf2-signalling did not show a significant change in muscle weakness (measured by exercise workload and 6-min walk test), while submaximal exercise heart rate and plasma lactate were significantly lowered [74]. The small cohort size (8–13 participants per dosing group), as well as the short treatment period (12 weeks), may be considered as reasons for not meeting the efficacy endpoint. On the other hand, reduction of the submaximal exercise heart rate and plasma lactate may be indicative of a relevant abrogation of mitochondrial pathology. However, there is a need for further investigations regarding omaveloxolone in mitochondrial myopathies.

A prominent activation of oxidative stress has also been identified in diseases resulting from defects in fatty acid metabolism, of which multiple Acyl-CoA dehydrogenation deficiency (MADD) and Short-Chain Acyl-CoA Dehydrogenase Deficiency (SCADD) have been relevantly studied in this regard. In both diseases, a profound upregulation of oxidative stress is found, while the cause of ROS production is not fully elucidated. Antioxidant treatment of patient-derived fibroblasts with either SCADD- or MADD-deficiency resulted in reduction of oxidative stress and prolonged longevity, emphasizing the role of oxidative stress in both diseases [75,76,77]. 

### 4.3. Oxidative Stress in Inflammatory Myopathies

As described above, there is a substantial interaction of ER-stress and oxidative stress, as misfolded proteins and the arising UPR are a major source of ROS production. The same applies for dysregulation of calcium homeostasis by means of a persistent calcium transfer from the ER to mitochondria. As ER-stress, as well as dysregulation, of calcium homeostasis have been considered central pathogenic mechanisms in Idiopathic Inflammatory Myopathies (IIM), several authors have proposed the resulting ROS production to be a relevant part of IIM pathology [78,79]. However, sufficient experimental evidence supporting this hypothesis is still missing.

## 5. Hypoxic Stress

The muscle requires continuous supplementation with oxygen in order to ensure its innate function in terms of motion, postural stabilization and breathing and thus consumes a relevant part of the oxygen ingested (for details, see Section 2). If the local needs for oxygen exceed the available resources, a lack of oxygen by means of hypoxia arises. As a result of hypoxic stress, a number of mechanisms are activated that are summarized as hypoxia stress response pathways. The central effector of hypoxia stress response is a family of transcription factors called hypoxia inducible factors (HIFs), with hypoxia inducible factor 1-alpha (HIF-1α) being the best studied member of this family. In normoxic conditions, the HIF transcription factors are subject to rapid degradation, while they are stabilized under hypoxia, translocate to the nucleus, and bind to a specific DNA sequence termed hypoxia response element (HRE) [169]. This results in the activation of multiple effector genes that modulate a variety of cellular processes, including angiogenesis and glucose metabolism, as well as fatty acid metabolism. Interestingly, there seems to be a relevant influence on HIF expression by other forms of cellular stress, including ER- and oxidative stress [170].

Given the prone importance of oxygen supplementation to ensure proper muscle function and integrity, there is an astonishing lack of information regarding hypoxia and the hypoxia stress response in muscular disorders. It has been demonstrated that even recurrent episodes of mild hypoxia are able to induce apoptosis and inflammation in skeletal muscle [171]. Consistently, chronic hypoxia has been identified as an important pathogenic mechanism of muscle injury secondary to other diseases, e.g., chronic obstructive pulmonary disease (COPD) [172]. 

As for primary muscle disorders, however, there is little information regarding the role of hypoxia in muscle pathology. This particularly surprising as several muscle disorders are known to cause chronic hypoxia due to hypoventilation as consequence of diaphragmatic weakness [173]. In Duchenne muscular dystrophy (DMD), several authors have proposed disease stage dependent alterations of the muscle microvasculature, both in patients, as well as mdx mice [80,81,82,83,84]. Further evidence of an impaired hypoxia stress response in DMD was gained by a publication suggesting an impaired ability of satellite cells to promote angiogenesis [85]. Finally, it has been demonstrated, that muscle function is relevantly impaired upon chronic hypoxia in a drosophila model of DMD [86]. Based on these observations, a relevant role of hypoxia stress response, as well as HIF-signaling, in DMD pathology has been hypothesized, although experimental evidence is missing to a great extent [174]. Notably, there is another muscular dystrophy that may partially rely on the dysregulation of hypoxic stress. A meta-analysis combining multiple microarray datasets from FSHD patients has yielded evidence of a perturbed HIF-signaling cascade, while sufficient experimental proof has not been gained to date [87]. 

Activation of the hypoxia stress response has further been identified in Idiopathic Inflammatory Myopathies (IIM). First evidences suggesting that patients with IIM suffer from a relevant muscular hypoxia have been gained in the late 1980s [88]. Since then, several studies have reported profound microvascular disturbances in patient samples of several forms of IIM (reviewed in Reference [89]). Consistently, it has been shown that major regulators of hypoxia stress response, including HIF-1α and IFN-β, are upregulated in IIM [90,91]. Interestingly, it appears as if there is a relevant difference between juvenile and adult forms of IIM. A recent study indicates that hypoxia-driven signaling pathways a preferentially activated in patient samples of juvenile IIM [92]. 

## 6. Mitochondrial Stress Response 

In the same way as the individual cell, mitochondria are faced with continuous perturbations of homeostasis that require an adaptive and coordinated reaction to overcome the respective stressor. In this concern, there has been growing evidence that mitochondria possess an innate set of response mechanisms to cope with a variety of stressors [175]. The whole ensemble of these mechanisms has been summarized as mitochondrial stress response and includes an mitochondria-specific unfolded protein response (UPR^mt^), as well as adaptive changes in amino acid and fatty acid metabolism (thoroughly reviewed in Reference [176]). While information regarding the mitochondrial stress response in the context mitochondrial myopathies in humans are sparse, several studies suggested a relevant activation of stress-related pathways in mouse models of mtDNA deletion diseases [93,94]. Notably, a recent publication has identified a concerted upregulation of the metabolic cytokines FGF21 and GDF15, as well as an activation of the UPR^mt^ and adaption of the one-carbon metabolism, in a mouse model of mitochondrial myopathy. This multimodal integrated mitochondrial stress response was demonstrated to rely on activation of mTOR-signalling cascade leading to ATF4-activation. Interestingly, treatment with the mTOR-inhibitor rapamycin was able to ameliorate the muscular pathology and to improve muscle weakness [95]. These results may offer a novel therapeutic approach for patients with mitochondrial myopathy due to multiple DNA deletions. However, further research is needed to extend our knowledge regarding mitochondrial stress response in the context of mitochondrial myopathy.

## 7. Integrated Stress Response and Stress Granule Formation

**Figure 4:**
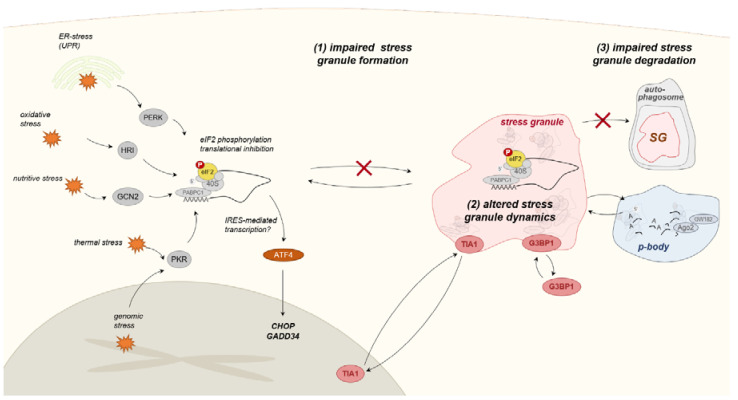
Schematic representation of the integrates stress response (ISR) in the context of muscular disorders. Stressor-specific response pathways converge into a unified pathway by means of ISR. Central mechanism of the ISR is the phosphorylation of eukaryotic translation initiation factor 2α (eIF2α), leading to the translational inhibition of the preinitiation complex. The non-translating complexes are incorporated into stress granules together with other RNA-binding proteins (e.g., TIA1 and G3BP1). In muscle diseases, both an impaired stress granule formation, as well as altered stress granule dynamics, have been identified as pathogenic mechanisms. Furthermore, a defective stress granule degradation via autophagic pathways has been considered to contribute to disease progression.

A stressor-specific response is induced as reaction to the respective disturbance of cellular homeostasis (e.g., protein misfolding, oxidative stress, hypoxia) in order to address the specific needs of the perturbation. However, they all converge into a unified pathway of stress response mechanisms termed the integrated stress response (ISR) [18]. ISR is believed to mediate the induction of general adaptive mechanisms that consolidate the cell in the context of cellular stress or—if the stress level exceeds the coping mechanisms—induce apoptotic pathways [177]. One of the central steps in ISR initiation is phosphorylation of the eukaryotic initiation factor 2 alpha (eIF2α) at a serine residue (Ser51) resulting in inhibition of translation initiation at the level of the preinitiation complex [178]. The untranslating mRNA-protein complexes (ribonucleoprotein complexes, RNPs) assemble into larger non-membranous cellular organelles (RNP-granules) termed stress granules [179]. Next to translationally silenced mRNPs, stress granules comprise a number of RNA-binding proteins (RBPs) that are either essential for stress granule formation and stability or optional constituents [180]. There is an ongoing debate regarding the function of stress granules in context of cellular stress. Discussed consequences include sequestration of specific molecules in stress granules lowering their concentration in the cytoplasm and thus influencing numerous translational and non-translational reactions. Furthermore, as result of the aggregation of preinitiation complexes, a preferential translation of specific mRNAs has been discussed, that may among others rely on internal ribosomal entry site (IRES)-mediated translation. Finally, the high local concentration of initiation factors may facilitate formation of the initiation complex even under conditions of cellular stress [179,181]. Despite the uncertainties regarding the specific roles of stress granules, inhibition of stress granule formation results in poorer survival rates upon application of cellular stress, emphasizing the importance of stress granules to the cellular stress response [181]. As described above, ISR results in a translational activation of specific factors that may be facilitated by stress granule formation. The perhaps most important transcription factor activated by ISR is activating transcription factor 4 (*ATF4*), mediating the expression of several downstream effectors, including *CHOP* [182]. This results in stimulation of both protective factors (e.g, chaperones and antioxidants), as well as pathways leading to apoptosis and cell-cycle arrest [177]. In context of muscular disorders, there has been increasing evidence that defects in central mechanisms of the ISR may have a pivotal role in the pathogenesis of several muscle diseases (Figure 4). 

Of specific interest in this concern are some disease entities from the group of distal myopathies. Distal myopathies are a heterogeneous group of rare neuromuscular disorders showing a predominant distal distribution of muscular weakness [183]. One member of distal myopathies is Welander distal myopathy (WDM), an adult-onset neuromuscular disorder that is characterized by predominant affection of wrist- and finger extensors [184]. WDM is caused by mutations in *TIA1*, a gene encoding for Tia1 cytotoxic granule-associated RNA binding protein [100]. *TIA1* is an essential component of stress granules, while its depletion results in impaired stress granule formation upon application of cellular stress [185]. In terms of WDM, *TIA1*-mutation did not lead to abolition of stress granule formation. However, recovery experiments suggested altered stress granule dynamics as a relevant factor in WDM pathogenesis [100]. Interestingly, mutations in *TIA1* are also considered a relevant co-factor in multisystem proteinopathy caused by sequestosome-1 (*SQSTM1*), a protein involved in autophagy [186]. This again emphasizes the interaction of cellular stress response and autophagic pathways. 

Another distal myopathy that has been associated to defects in ISR is Matrin-3-associated distal myopathy (MATR3-myopathy). MATR3-myopathy is a distal myopathy clinically characterized by weakness of foot dorsiflection, finger and wrist extension, as well as early axial affection [187,188]. Furthermore, dysphonia and dysphagia, as well as restricted ventilation, are typically seen in advanced disease stages [188]. Cytoplasmic aggregates of the stress granule proteins *TIA1* and Ras GTPase-activating protein-binding protein 1 (*G3BP1*) are observed in muscle biopsy specimen of MATR3-myopathy, suggestive of defects in stress granule formation or dynamics. Consistently, an impaired stress granule formation in patient-derived primary fibroblasts with *MATR3*-mutation was identified, leading to reduced cellular vitality upon application of cellular stress [97]. Although the underlying mechanisms remain elusive, the results emphasize the role of ISR and stress granule formation in the pathogenesis of MATR3 myopathy.

Furthermore, a distinct form of distal myopathy can arise from mutations in VCP [98]. While VCP is a multifunctional protein, for example involved in UPR and autophagy, it has also been identified as a regulator of stress granule assembly [99]. However, the functional abrogation caused by the specific mutation leading to VCP-associated distal myopathy have not been studied in detail. Thus, there can only be speculated regarding an involvement of ISR in VCP-associated distal myopathy.

Apart from classical distal myopathies, impaired stress granule formation has also been identified as a relevant pathogenic factor in myotonic dystrophy type 1 (DM1), a DNA-expansion disorder caused by expanded CTG repeats in the *DMPK* gene. Patients with DM1 suffer from progressive muscular weakness with a predominant distal phenotype, as well as myotonia, early-onset cataracts, diabetes mellitus, and variable cognitive impairments [189]. Defects in splicing by sequestration of splicing related RBPs have been identified as the core pathogenetic mechanism of DM1. However, a recent publication identified a muscle-specific upregulation of Staufen 1 leading to disruption of stress granule formation, while depletion of Staufen 1 resulted in restoration of stress granule assembly [96].

## 8. Conclusions

Based on its prone dependence on a rapid and coordinated adaption to changes in cellular homeostasis, there is an evident need of the muscle for a fully functional and effective cellular stress response. In this concern, it is of no surprise that deregulation of the cellular stress response has been identified in several muscular disorders. There is emerging evidence for a relevant contribution of the cellular stress response to the pathophysiology of both hereditary (e.g., muscular dystrophies and mitochondriopathies) and acquired (e.g., inflammatory myopathies and myasthenia gravis) muscle disorders. The defects observed may affect both the stressor-specific adaptive response, as well as the integrated stress response as the final common path. While a small subset of muscular disorders, including DMD and IBM, have been studied with comparable intense, detailed information regarding underlying mechanisms is lacking for most of the diseases. Based on the current evidence, deregulation of cellular stress response may arise as a secondary consequence of the primary pathogenetic mechanism in most of the muscle disorders. Nonetheless, the deregulated cellular stress response must be considered a relevant sustentative factor in pathogenesis, leading to a vicious circle. Furthermore, a small subset of muscle disorders seems to primarily rely on defects of relevant factors (e.g., chaperones, stress granule components) or mechanisms (stress granule formation) of the cellular stress response. In this scenario, a deregulated cellular stress response is likely to be the primary cause of the disease.

Irrespective of being cause or consequence, deregulation of the cellular stress response appears to relevantly add to disease progression. Several approaches to ameliorate defective stress response mechanisms in respective disease models have resulted in a relevant improvement of cellular function and muscle pathology. However, while some preclinical results regarding cellular stress response as therapeutic target yielded promising results, most of the few clinical trials conducted were not able to identify beneficial effects. The reasons for the lack of sufficiency may be diverse, including an unfavorable study design (small cohorts, short duration), as well as tissue- and species-specific issues (e.g., low local concentrations of the active agent). Furthermore, the results gained in individual disease models might not fully account for the complexity of the genuine muscle disease. Hence, there is an immanent need for further studies addressing both underlying mechanisms, as well as the resulting consequences, in detail and thus profoundly improving the current knowledge regarding the cellular stress response in muscular disorders.

## Figures and Tables

**Table 1:** Muscular diseases with deregulated cellular stress response.

Muscular Disease	Gene	Protein	Reference
**ER-stress/UPR**			
**Muscular dystrophies**			
Caveolinopathy (LGMD1C/RMD2)	CAV3	caveolin-3	[19]
Duchenne muscular dystrophy (DMD)	DMD	dystrophin	[20,21,22]
GNE myopathy (hIBM2, Nonaka myopathy)	GNE	Bifunctional UDP-*N*-acetylglucosamine 2-epimerase/*N*-acetylmannosamine kinase	[23,24]
Limb girdle muscular dystrophy 1E (LGMD1E/LGMDD1)	DNAJB6	dnaJ homolog subfamily B member 6	[25,26,27]
Limb girdle muscular dystrophy 2I (LGMD2I/LGMDR9)	FKRP	fukutin-related protein	[28,29]
Tibial muscular dystrophy (TMD, Udd myopathy)	TTN	titin	[30]
**Myofibrillar Myopathies**			
Myofibrillar myopathy-2 (MFM2)	CRYAB	alpha-crystallin B chain	[25,31]
Myofibrillar myopathy-6 (MFM6)	BAG3	BAG family molecular chaperone regulator 3	[25,32]
**Congenital Myopathies**			
Congenital myopathy with fiber-type dysproportion	SELENON	selenoprotein N	[33]
Central core myopathy	RYR1	ryanodine receptor 1	[34]
**Metabolic Myopathies**			
Glycogen storage disease II (GSD2, Pompe disease)	GAA	lysosomal alpha-glucosidase	[35]
Lipin 1-myopathy (acute recurrent myoglobinuria, autosomal recessive)	LPIN1	phosphatidate phosphatase LPIN1	[36]
**Idiopathic inflammatory Myopathies (IIM)**			
IIM in general			[37,38,39]
Sporadic inclusion body myositis (sIBM)			[40,41,42,43,44,45,46,47,48,49]
**Other Muscle Disorders**			
Myasthenia gravis			[50,51]
**Oxidative Stress**			
**Muscular Dystrophies**			
Duchenne muscular dystrophy (DMD)	DMD	dystrophin	[52,53,54,55,56,57,58,59,60,61]
Dysferlinopathy (LGMD2B/LGMDR2)	DYSF	dysferlin	[62]
Facioscapulohumeral muscular dystrophy (FSHD)	D4Z4/DUX4	–	[63,64,65,66,67]
GNE myopathy (hIBM2, Nonaka myopathy)	GNE	Bifunctional UDP-N-acetylglucosamine 2-epimerase/*N*-acetylmannosamine kinase	[68]
**Mitochondriopathies**			
mtDNA-associated diseases (e.g., CPEO, MELAS, MERRF)	mtDNA	-	[69,70,71,72,73,74]
multiple Acyl-CoA dehydrogenation deficiency (MADD)	ETFDH	electron transfer flavoprotein-ubiquinone oxidoreductase, mitochondrial	[75]
short-chain Acyl-CoA dehydrogenase deficiency (SCADD)	ACADS	short-chain specific acyl-CoA dehydrogenase, mitochondrial	[76,77]
**Idiopathic Inflammatory Myopathies (IIM)**			
IIM in general			[78,79]
**Hypoxic Stress**			
**Muscular dystrophies**			
Duchenne muscular dystrophy (DMD)	DMD	dystrophin	[80,81,82,83,84,85,86]
Facioscapulohumeral muscular dystrophy (FSHD)	D4Z4/DUX4	-	[87]
**Idiopathic Inflammatory Myopathies (IIM)**			
IIM in general			[88,89,90,91,92]
**Mitochondrial Stress Response**			
mtDNA-associated diseases (e.g., CPEO, MELAS, MERRF)	mtDNA	-	[93,94,95]
**Integrated Stress Response**			
Myotonic dystrophy type I (DM1)	DMPK	myotonin-protein kinase	[96]
MATR3-associated distal myopathy (MPD2, VCPDM)	MATR3	matrin-3	[97]
VCP-associated distal myopathy	VCP	transitional endoplasmic reticulum ATPase	[98,99]
Welander distal myopathy (WDM)	TIA1	nucleolysin TIA-1 isoform p40	[100]
